# Metabolomics analysis reveals the metabolic and functional roles of flavonoids in light-sensitive tea leaves

**DOI:** 10.1186/s12870-017-1012-8

**Published:** 2017-03-20

**Authors:** Qunfeng Zhang, Meiya Liu, Jianyun Ruan

**Affiliations:** 1 Tea Research Institute, Chinese Academy of Agricultural Sciences, Hangzhou, 310058 China; 2 Key Laboratory for Plant Biology and Resource Application of Tea, the Ministry of Agriculture, South Meiling Road 9, Hangzhou, Zhejiang 310008 China

**Keywords:** *Camellia sinensis*, Flavonoids, Light sensitive, Metabolism, Photo-protection

## Abstract

**Background:**

As the predominant secondary metabolic pathway in tea plants, flavonoid biosynthesis increases with increasing temperature and illumination. However, the concentration of most flavonoids decreases greatly in light-sensitive tea leaves when they are exposed to light, which further improves tea quality. To reveal the metabolism and potential functions of flavonoids in tea leaves, a natural light-sensitive tea mutant (*Huangjinya*) cultivated under different light conditions was subjected to metabolomics analysis.

**Results:**

The results showed that chlorotic tea leaves accumulated large amounts of flavonoids with ortho-dihydroxylated B-rings (e.g., catechin gallate, quercetin and its glycosides etc.), whereas total flavonoids (e.g., myricetrin glycoside, epigallocatechin gallate etc.) were considerably reduced, suggesting that the flavonoid components generated from different metabolic branches played different roles in tea leaves. Furthermore, the intracellular localization of flavonoids and the expression pattern of genes involved in secondary metabolic pathways indicate a potential photoprotective function of dihydroxylated flavonoids in light-sensitive tea leaves.

**Conclusions:**

Our results suggest that reactive oxygen species (ROS) scavenging and the antioxidation effects of flavonoids help chlorotic tea plants survive under high light stress, providing new evidence to clarify the functional roles of flavonoids, which accumulate to high levels in tea plants. Moreover, flavonoids with ortho-dihydroxylated B-rings played a greater role in photo-protection to improve the acclimatization of tea plants.

**Electronic supplementary material:**

The online version of this article (doi:10.1186/s12870-017-1012-8) contains supplementary material, which is available to authorized users.

## Background

Flavonoids, the main secondary metabolites in plants, are the most important quality-related compounds, and they comprise 20–40% of the dry matter in young shoots of tea plant [1]. These flavonoid compounds also contribute to the color, taste and aroma of brewed tea. The contents of catechins (flavan-3-ol), flavonols, flavonol glycosides, anthocyanin and leucoanthocyanidin account for the majority of flavonoids in tea plant. The basic molecular structure / carbon skeleton of flavonoids is C6-C3-C6, and they are classified as mono-, di- and tri-hydroxylated based on their hydroxylated B-rings. The synthesis and accumulation of flavonoids occur in response to environmental cues [2]. Numerous studies have shown that the biosynthesis of flavonoids (expression of structural genes, activity of some important enzymes and concentrations of flavonoids) increases under high light intensity [2, 3]. However, sub-groups of flavonoids in tea plants can be differentially affected [4, 5]. The fundamental role of flavonoids to cope with light stress may rely on their many potential functions [6].

The flavonoids metabolic pathway has been widely accepted to be involved in the regulation mechanisms of plants to various stressful conditions [7]. Flavonoids are the main regulators of plant growth and defense, and they are induced and biosynthesized as the result of a long-term natural selection and acclimatization process [8–12]. The main physiological functions of flavonoids in tea plant are scavenging reactive oxygen species and increasing tolerance to adapt to environmental change, e.g., as antioxidants in photoprotection. The antioxidant activity of flavonoids is attributed to their reaction with free radicals as a hydrogen donor. However, flavonoids with different molecular structures have great differences in their antioxidant activity, which is highly correlated with the substituent positions and the amount of hydroxyl groups on the B-rings. The more hydroxylation of the flavonol, the more hydrogen atoms can be provided for binding free radicals, and the more antioxidant activity; e.g., the antioxidant activity of myricetin is higher than that of kaempferol. Moreover, the tautomeric interconversions of ortho-dihydroxylated B-ring flavonoids make them more efficient at dissipating excess energy [13] and scavenging reactive oxygen species [6, 14, 15].

*Camellia sinensis* (L.) O. Kuntze cv. *Huangjinya*, a natural light-sensitive tea mutant, shows high levels of free amino acids and a low content of polyphenols, which improves the quality of brewed green tea and imparts a higher economic value to the chlorotic varieties than to the non-chlorotic varieties [16–19]. The young leaves of *Huangjinya* display a yellow color and show normal growth despite being chlorotic under high light conditions [20]. Moreover, *Huangjinya* shows a totally different metabolic response to light compared to the normal tea species. For example, as the main type of secondary metabolism in tea plant, the biosynthesis of flavonoids increases with increasing temperature and illumination [5]. However, the concentration of most flavonoids decreases greatly in *Huangjinya* leaves when they are exposed to light. The characteristics of specific genes and chemicals such as total polyphenols, total amino acids, and pigments biosynthesis in *Huangjinya* has been reported in the previous studies [17, 20]. Although less attention has been paid to the metabolic regulation of flavonoids in this mutant, such an analysis may help to clarify the mechanisms by which flavonoids highly accumulate in tea plants.

Light is one of the most important environmental factors, providing energy and external stimuli for growth and development in plants [21]. Changes in light intensity alter a complex set of molecular events within the chloroplast [5, 22]. However, light energy beyond the acceptable range of the reaction center causes light damage, leading to photoinhibition and decreasing the photochemical and carbon assimilation rates [23, 24]. Thus, photoprotection, either by scavenging harmful reactive intermediates or dissipating excess energy to protect cells from death under light stress, is essential for photosynthesis [25]. Chloroplast-located flavonoids [10, 26], phenylpropanoids [27] and anthocyanins [28] are effective sunlight attenuators which play vital roles in protecting chloroplasts when facing with a superabundant radiant energy. The key antioxidant function of flavonoids to excess ultraviolet radiation b (UV-B)/sunlight irradiance have been well documented [29–31]. These studies are further confirmed by the fact that flavonoids with an ortho-dihydroxy structure in the B-ring are preferentially accumulated, compared to flavonoids having a mono-hydroxy substitution [32, 33]. The carotenoid or xanthophyll cycle also participates in protecting plants from high light intensity by thermal dissipation [34]. Moreover, organisms show chlorosis when the carotenoid biosynthesis pathway is disrupted [25] or is inhibited by norflurazon [35].

Herein, the hypothesis of the present work was that the metabolism of flavonoids, especially the ‘different branch’, may be regulated by genetic and environmental stimuli in light-sensitive tea leaves and that it is highly correlated with the potential function of flavonoids in tea leaves. An experiment was therefore designed in which samples of tea leaves from a light-sensitive mutant cultivated under two different light conditions were compared using metabolomics analysis.

## Methods

### Plant material and shading treatment

Rooted-cuttings of the natural mutant *Camellia sinensis* (L.) cv. *Huangjinya*, which was officially released as a clone in Zhejiang province in 2008 were obtained free of charge from the owner of the mutant Yuyao Deshi Tea Plantation (located in Yuyao county, Zhejiang province). Each of four seedlings were planted in pots (~10 L capacity) filled with commercial growth medium consisting of perlite, vermiculite, and peat at the Tea Research Institute, Chinese Academy of Agricultural Sciences (TRI, CAAS, N 30°10′, E 120°5′) in May of 2013. The pots were placed in open with full sunlight and watered regularly. In March 2014, 60 pots of tea plants with uniform young shoots were selected for the experiment: Half of the pots were treated with high-density polyethylene tape two-pin net (60% sun-shading), and the remaining 30 pots were exposed to full sunlight for ten days. Samples (young shoots with bud and two expanding leaves) were randomly selected, frozen with liquid nitrogen and then stored in a −70 °C ultra-freezer. The sampling was repeated six times each from shaded and unshaded plants.

### Ultra-performance liquid chromatography quadrupole time of flight mass spectrometry (UPLC-Q-TOF/MS) based metabolomics analysis

All 12 obtained samples (six biological repetitions for the shaded and unshaded groups) were used for metabolomics analysis. The metabolites were extracted from young shoots using a mixture of 75% methanol and 1% formic acid as described by Zhang et al. [5]. A 2 μL sample was injected into a UPLC-Q-TOF/MS (Waters, UPLC/Xevo G2-S Q-TOF) and separated with an HSS T3 column as described by Zhang et al. [5]. TransOmics software (Version 1.0; Waters) was applied for data preprocessing. Metabolite peaks were assigned to the accurate mass measurements using online metabolite databases as described in Zhang et al. [5], and the retention times were compared with those in the published literature [5]. A matrix was exported for further statistical analyses. The unit variance was scaled for further statistical analysis using SIMCA-P (version 13.0, Umetrics, Umea, Sweden). Unsupervised principal component analysis (PCA) and supervised projection to latent structure discriminant analysis (PLS-DA) were carried out to dissect the overall variance of metabolites and the composition differences of the samples, respectively. The combination of p(corr) and variable importance in the projection (VIP) values from the PLS-DA were used as a coefficient for metabolite selection (VIP > 1.0 and |p(corr)| > 0.5). Student’s *t*-test (*P* < 0.05) and one-way analysis of variance (ANOVA) using the SPSS (version 15.0, SPSS Inc., Chicago, IL) were performed for statistical analysis.

### Quantitative real-time PCR analysis

Total RNA was isolated using a plant RNA extraction kit (Tiangen, China). PrimeScriptTM RT reagent kit (TaKaRa) was applied to synthesize cDNA. Quantitative real-time PCR (qRT-PCR) was performed on the Applied Biosystems 7300 machine (Carlsbad, CA, USA). The primer pairs used for qRT-PCR are shown in Additional file 1: Table S1, and *GAPDH* was used as the reference gene. For each target gene, triplicate reactions were performed. Relative transcript levels were calculated against that of the internal control *GAPDH* using the formula 2^-ΔΔCt^. All data are shown as the mean ± SD (*n* = 3).

### Quantitative determination of chlorophylls, carotenoids, and catechins

To determine the contents of chlorophyll and carotenoids, leaf discs with an area of 86.59 mm^2^ were removed using a perforated metallic cylinder. Tea infusions were analyzed on a reverse phase high-performance liquid chromatographic (HPLC) system (Waters 2695) coupled to a diode array detector (Waters 2998) as described in [5]. Catechins were also quantified by HPLC, and the separations were performed using a C18 reverse-phase column (250 × 4.6 mm i.d., Phenomenex, Torrance, CA, USA) as described by Wu et al. [36].

### Tissue localization of phenolic compounds

Samples prepared by standard freehand sectioning and stained with 1% (*w/v*) vanillin-HCl reagent were used to study the localization of phenolic compounds. Photos of the sample sections were taken by a microscope (XQT-2, COIC) before and after staining [37].

The localization of flavonoids was determined by staining the sections with NaturstoVreagenz A and observing using confocal laser scanning microscopy (CLSM, Zeiss LSM 710 NLO) by the method described in [38].

## Results

### Phenotype and content of chlorophyll and carotenoids in Huangjinya leaves

**Fig. 1:**
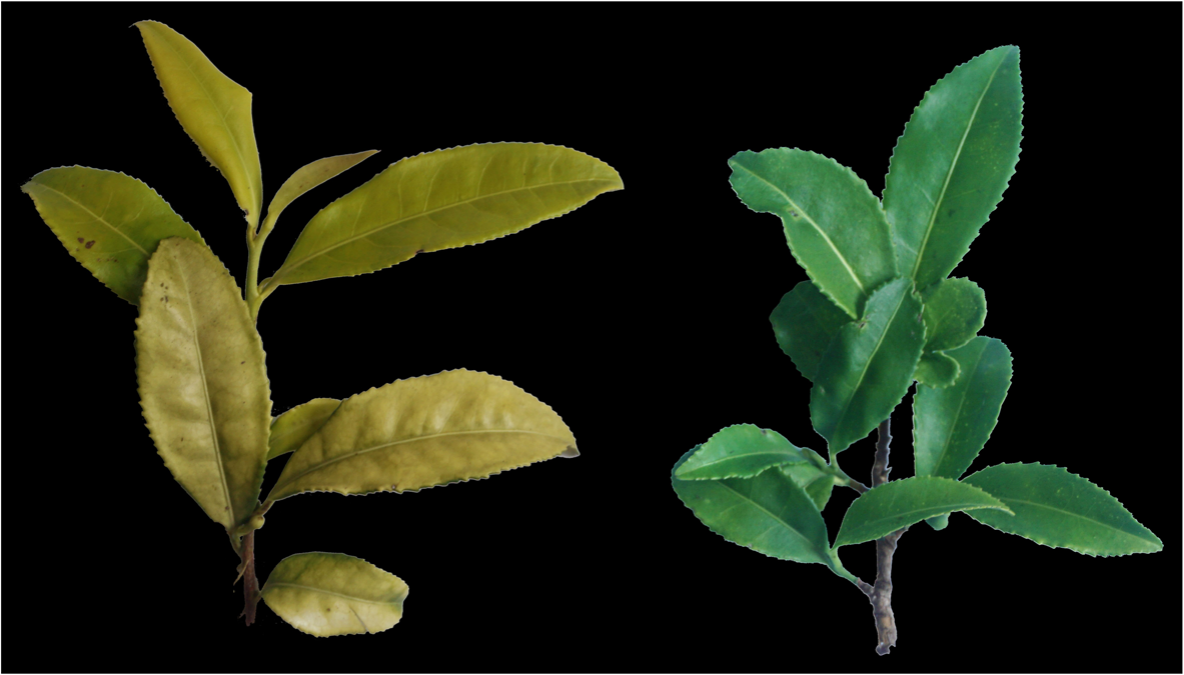
Phenotypic characterization of chlorotic and green leaves under full sunlight (*left*) and shaded (*right*, 60% light intensity) conditions

As shown in Fig. 1, the leaves were chlorotic when the tea plants were exposed to full sun light condition, whereas the leaves of shaded tea plants turned green. Accordingly, the contents of chlorophyll in these two types of tea plant leaves were distinct (Table 1). Compared to the non-chlorotic tea plants, chlorotic leaves had a relatively lower levels of chlorophyll-a and chlorophyll-b. As the light-harvesting pigments in plant photosynthesis system, carotenoids ensure efficient photosynthesis and prevent light damage for plants. In our study, the contents of lutein and neoxanthin reduced and with an increment of zeaxanthin and carotene in the chlorotic leaves (Table 1).

**Table 1:** Concentrations of the main catechins and carotenoids in young shoots of chlorotic (full sunlight) and green (shaded) tea plants

Name	Shaded green (G) plants	Chlorotic (C) plants
Catechins (mg/g fresh weight)		
Catechin gallate	0.01 ± 0.00a	0.06 ± 0.01b
Gallate acid	0.09 ± 0.03	0.16 ± 0.03
Gallocatechin	0.26 ± 0.04a	0.08 ± 0.01b
Epicatechin	1.73 ± 0.12a	2.36 ± 0.21b
Catechin	0.08 ± 0.02	0.10 ± 0.01
Epigallocatechin	0.59 ± 0.04a	0.72 ± 0.04b
Gallocatechin gallate	0.29 ± 0.05	0.24 ± 0.06
Epigallocatechin gallate	9.32 ± 0.51a	7.82 ± 1.1b
Epicatechin gallate	5.24 ± 0.23	5.34 ± 0.21
Carotenoids and chlorophyll (μg/cm^2^ leaf area)		
Neoxanthin	16.44 ± 0.17a	1.21 ± 0.03b
Lutein	50.90 ± 3.39a	11.15 ± 0.12b
Violaxanthin	3.45 ± 0.11a	1.78 ± 0.06b
β-Carotene	8.08 ± 0.14a	13.02 ± 0.81b
Zeaxanthin	3.22 ± 0.17a	10.12 ± 0.19b
Chlorophyll-a	128.84 ± 1.31a	5.83 ± 0.16b
Chlorophyll-b	34.46 ± 2.26a	9.01 ± 0.12b

Different letters following data of the same lines indicate significant difference at *p* < 0.05

### Overview of metabolomic profiling

**Fig. 2:**
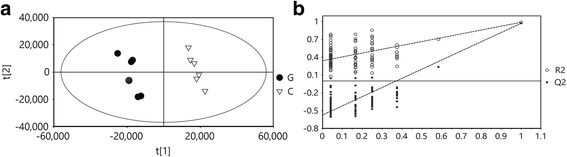
PLS-DA score plots (**a**) and permutation test (**b**) of metabolites analyzed by UPLC-Q-TOF/MS in young tea shoots

A total of 1471 compounds were extracted from the raw data of the UPLC-TOF/MS analysis. We performed PLS-DA modeling to determine which metabolites were significantly affected by chlorosis (Fig. 2a). The components of the PLS-DA model explained 89.3% variance and the cumulative Q2 variance was 82.2% for the prediction accuracy, indicating the inter-group difference is significant. PLS-DA models were further confirmed by a 7-fold cross validation, and a permutation test was applied to validate the models’ reliability rigorously (Fig. 2b, *n* = 200). The score plots showed a clear separation between the chlorotic and the shaded green leaves with the first component (shown as the X axis in Fig. 2a). Differential metabolites were identified based on the VIP > 1.0 and |p(corr)| > 0.5 in the PLS-DA model and *p* < 0.05 in the *t*-test. Fifty-two differential metabolites were identified, which were responsible for flavonoid biosynthesis and phenylalanine metabolism (Table 2). These metabolites include flavan-3-ol (catechins), flavonols, flavonol glycosides, anthocyanin, and benzoic acid and derivatives.

**Table 2:** Significantly changed (VIP > 1 and |p(corr)| > 0.65 from partial least squares discriminant analysis) intercellular metabolites induced by chlorosis

Compounds	VIP^a^	p(corr) ^b^	Log2 (C/G) ^c^
(−)-Epicatechin 8-C-glucoside	1.57	−0.97	−1.86
2,6-Digalloylglucose	1.58	−0.97	−1.08
3-(4-Hydroxybenzoyl) epicatechin	1.52	−0.94	−1.27
6-Galloylglucose	1.48	−0.88	−2.10
Anthocyanidins	1.54	0.95	2.10
Catechin 7-O-apiofuranoside	1.35	0.84	0.75
Catechin-galacto	1.38	−0.86	−0.48
Cyanidin 3-xyloside	1.25	0.78	1.30
Delphinidin 3-glucoside	1.16	−0.72	−1.64
Dihydromyricetin	1.51	0.93	2.53
Gallocatechin 3-gallate	1.60	−0.99	−1.17
Epicatechin 3-O-(3-O-methylgallate)	1.54	−0.96	−1.69
Epicatechin-ent-epicatechin	1.24	−0.77	−2.49
Catechingallate	1.10	0.69	2.37
Epigallocatechin	1.38	0.87	0.68
Kaempferol 3-(2G-apiosylrobinobioside)	1.43	−0.88	−1.58
Kaempferol 7-(6″-galloylglucoside)	1.59	0.99	1.38
Kaempferol-3-O-glucoside	1.48	0.93	1.43
Kaempferol-3-O-rutinoside	1.52	−0.95	−0.59
Leucodelphinidin	1.45	0.89	1.54
Myricetin	1.27	−0.78	−1.22
Myricetin 3-(3″-galloylrhamnoside)	1.35	−0.82	−0.37
Myricetin 3,3′-digalactoside	1.58	−0.97	−1.03
Myricetin 3-arabinoside	1.29	−0.80	−1.73
Naringenin-4′-O-glucuronide	1.55	0.95	1.33
Naringin	1.54	0.93	1.49
Procyanidin C1	1.41	−0.87	−1.21
Quercetagetin 3′-methylether 7-glucoside	1.55	0.94	1.81
Quercetin	1.63	0.99	1.32
Quercetin 3-(3R-glucosylrutinoside)	1.57	0.97	1.59
Quercetin 3,7,4′-O-triglucoside	1.60	0.99	1.24
Quercetin 3-arabinoside	1.54	0.95	2.42
Quercetin 3-galactoside	1.63	0.99	0.48
Quercetin 3-O-alpha-D-arabinopyranoside	1.61	0.99	1.63
Quercetin 3-rutinoside 7-galactoside	1.53	0.95	4.35
Quercetin 4′,7-diglucoside	1.64	1.00	1.25
Quercetin-3′-glucuronide	1.63	0.99	1.21
Rutin	1.60	0.99	1.35
Cinnamic acid	1.63	0.99	0.85
Chicoric acid	1.53	−0.92	−2.17
Fertaric acid	1.53	−0.94	−1.25
p-Coumaric acid	1.29	−0.81	−0.73
Benzoic acid	1.77	0.92	0.81
Gallic acid	1.50	0.91	1.02
4-Hydroxybenzoic acid	1.38	0.85	1.72
Glucogallina	1.55	−0.79	−0.25
Chalcones	1.46	0.94	1.24
Caffeic acid	1.37	−0.90	−2.13
Frerulic acid	1.24	0.87	0.84
Quinine	1.77	0.92	3.04
Quinic acid	1.34	−0.83	−1.73
Shikimic acid	1.16	−0.73	−0.32

^a^ VIP means variable importance in the projection values from partial least squares discriminant analysis (PLS-DA)

^b^p(corr) indicates loadings scaled as a correlation coefficient (ranging from −1.0 to 1.0)

^c^C/G means the ratio of the mean peak intensity in chlorotic (C, full sun light) and green (G, shaded) tea plants

### Differences in flavonoid metabolism between chlorotic and normal green leaves

**Fig. 3:**
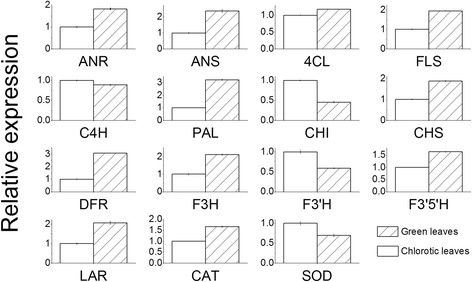
Quantitative RT-PCR validation. PAL, phenylalanine ammonia-lyase; C4H, cinnamate 4-hydroxylase; 4Cl, 4-coumarate--CoA ligase; CHI, chalcone isomerase; CHS, chalcone synthase; F3′5′H, flavonoid 3′, 5′-hydroxylase; F3H, flavanone 3-hydroxylase; F3′H, flavonoid 3′-monooxygenase; FLS, flavonol synthase; DFR, dihydroflavonol-4-reductase; ANR, anthocyanidin reductase; ANS, anthocyanidin synthase; LAR, leucoanthocyanidin reductase; CAT: catalase; SOD, superoxide dismutase

The chlorotic mutant showed lower expression levels of genes such as *Phenylalanine ammonia-lyase (PAL)*, *4-coumarate--CoA ligase* (*4Cl*), *anthocyanidin reductase* (*ANR*), *chalcone synthase* (*CHS*), *flavanone 3-hydroxylase (F3H), flavonoid 3′, 5′-hydroxylase (F3′5′H)* and *flavonol synthase (FLS)* than did the shaded green leaves in the pathway from phenylalanine to epigallocatechin. However, *chalcone isomerase (CHI)* and *flavonoid 3′-hydroxylase* (*F3′H*, the key gene of dihydroxy flavonoid synthetic) were up-regulated (Fig. 3) in the chlorotic leaves.

Analysis of metabolomics using UPLC-Q-TOF/MS revealed that the concentrations of flavan-3-ol, flavonols, and flavonol glycosides were greatly affected by shading (Table 2). Fifteen metabolites annotated as part of flavonoid metabolism were decreased in the chlorotic leaves compared with the green leaves, and these metabolites included (−)-epicatechin 8-C-glucoside, 6-galloylglucose, and epicatechin-ent-epicatechin. However, other flavonoids were not significantly reduced (e.g., epicatechin gallate, catechin) or were even increased (e.g., anthocyanidins, rutin, and catechin gallate) in the chlorotic leaves. It is interesting to note that the chlorotic leaves contained a higher level of dihydroxyflavonoids, mainly as quercetin and its glycosides (quercetin 3-galactoside, quercetin 4′,7-diglucoside, quercetin 3-rutinoside 7-galactoside etc.) than the green leaves, whereas the chlorotic leaves possessed a lower level of myricetrin glycoside (myricetin 3-arabinoside, myricetin 3,3′-digalactoside etc.) than did the green leaves (Table 2).

In the validation of quantitative analysis using HPLC, the chlorotic leaves showed lower contents of trihydroxy catechins (particularly the gallocatechin, epigallocatechin gallate) compared to shaded green leaves. However, the contents of dihydroxy catechins (catechin gallate, epicatechin) were significantly higher in the chlorotic leaves than in the shaded green shoots (Table 1).

Furthermore, metabolites involved in the metabolism of benzenoids, phenylpropanoids and polyketides, the branch upstream of catechin and flavonoid metabolism, were significantly downregulated in the chlorotic leaves (Table 2). Such as coumaric acid, caffeic acid, quinic acid, shikimic acid and chicoric acid.

### The intracellular localization of flavonoids and Gene expression related to the antioxidant system in chlorotic tea leaves

**Fig. 4:**
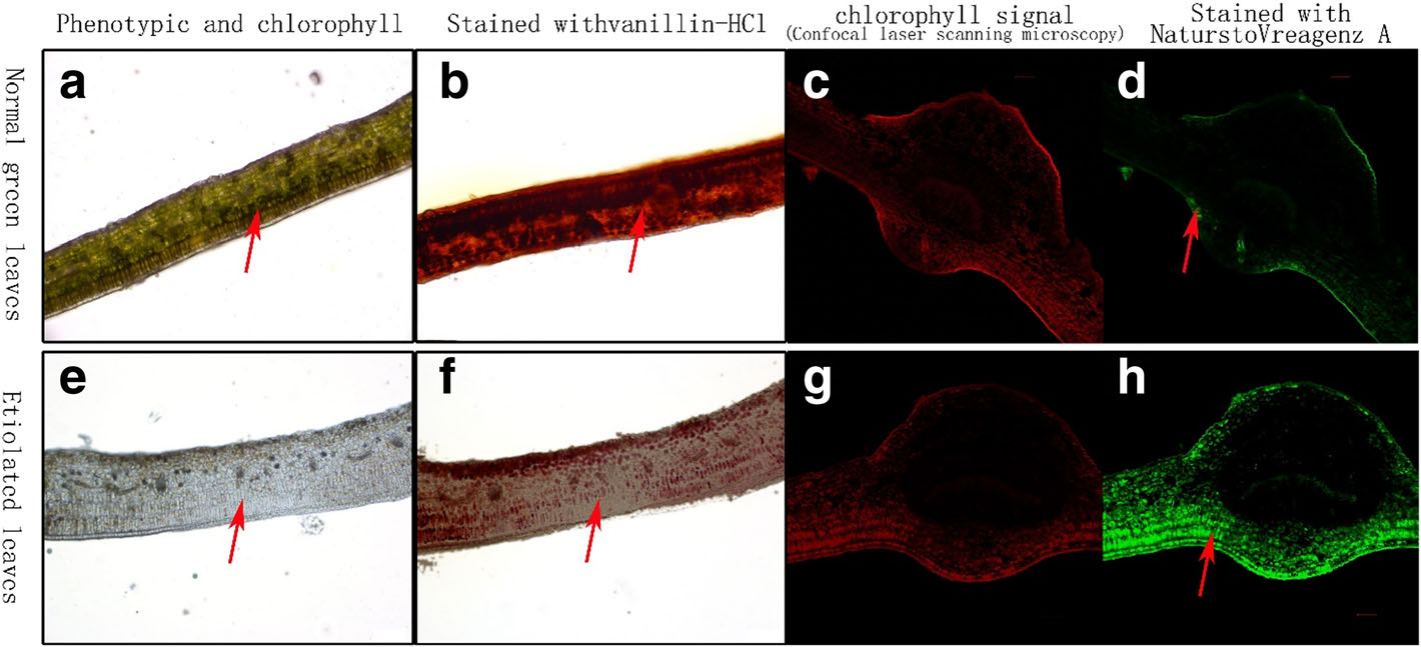
Histochemical localization of catechin and flavonol in *Huangjinya* leaves. **a**, **b**, **c**, **d** show the normal green leaves of *Huangjinya* shaded with 60% (NG); **e**, **f**, **g**, **h** show the chlorotic leaves of HJY exposed to 100% sunlight (EM); **b** and **f** show staining with vanillin-HCl reagent; the red signal represents phenolic compounds (mainly catechins; **d** and **h** were stained with NaturstoVreagenz A (confocal laser scanning microscopy), and the green fluorescence signal represents flavonol

The intracellular localization of flavonoids (mainly dihydroxy flavonoids) was highly different between the chlorotic and shaded green leaves (Fig. 4). The flavonoids (Fig. 4, green fluorescent signal) were mainly located in the epidermal leaf cells and in the light-receiving area of leaves, whereas chlorophyll (green and red signal in Fig. 4) and catechins (Fig. 4, red signal) were distributed throughout the cells of the shaded green leaves. However, the catechin signal (Fig. 4, red signal) was significantly weakened, the chloroplasts degraded (Fig. 4, arrows), and the dihydroxy flavonoids spread to all leaf cells as the leaves became chlorotic.

The expression level of the gene encoding catalase (CAT) was 1.4-fold higher in shaded green leaves than in chlorotic leaves (Fig. 3). By contrast, the gene encoding superoxide dismutase (SOD) were repressed in chlorotic leaves.

## Discussion

**Fig. 5:**
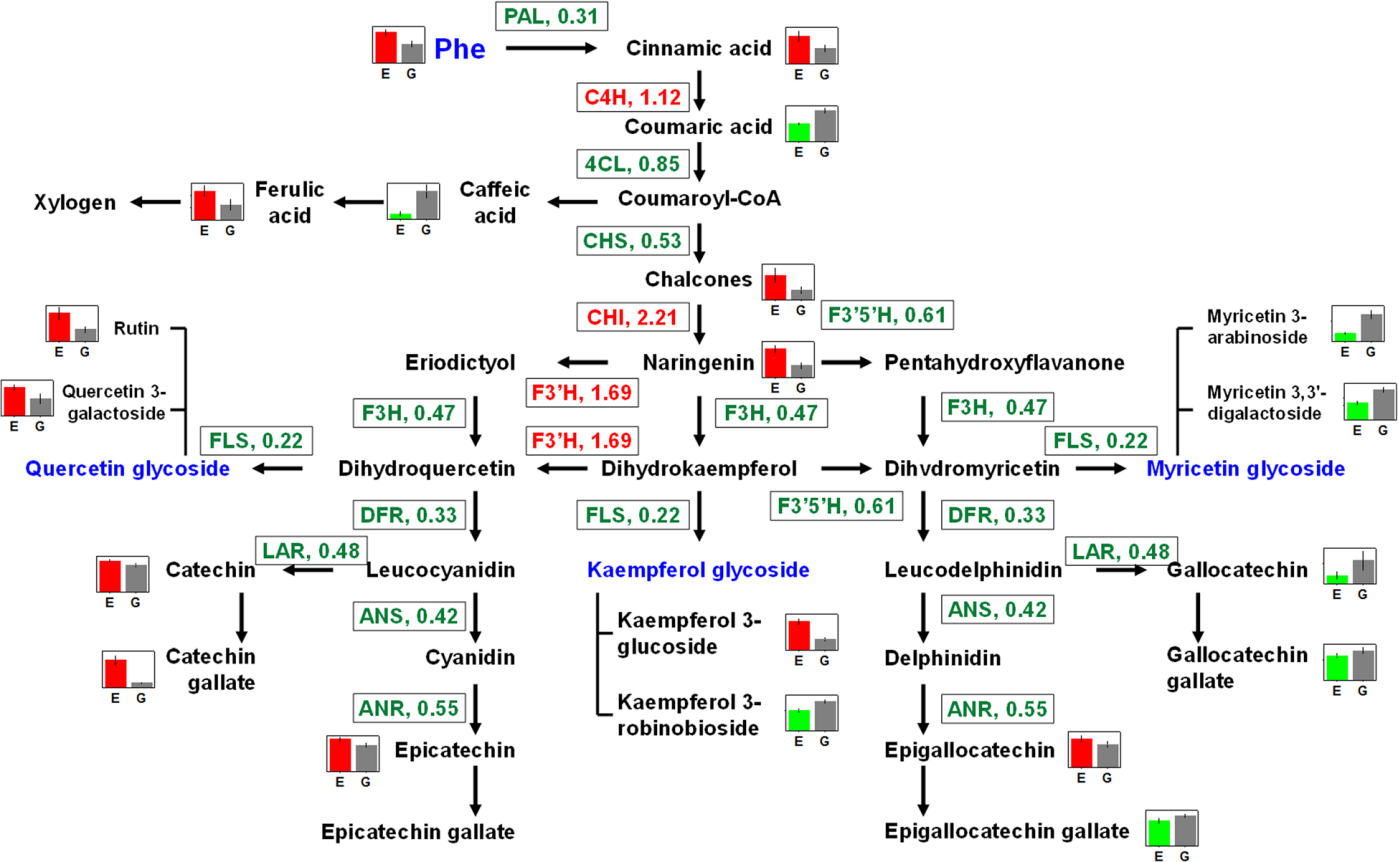
Schematic representation of phenylpropanoid/flavonoid pathway as affected by the chlorosis mutation. *Red* and *green* font indicate up- and down-regulated genes, respectively, in the chlorotic leaves compared to shaded plants. Significantly changed metabolites are shown with a barplot (E and G indicate chlorotic and green leaves, respectively) while compounds that were not identified in this study are shown in gray font. PAL, phenylalanine ammonia-lyase; C4H, cinnamate 4-hydroxylase; 4Cl, 4-coumarate--CoA ligase; CHI, chalcone isomerase; CHS, chalcone synthase; F3′5′H, flavonoid 3′, 5′-hydroxylase; F3H, flavanone 3-hydroxylase; F3′H, flavonoid 3′-monooxygenase; FLS, flavonol synthase; DFR, dihydroflavonol-4-reductase; ANR, anthocyanidin reductase; ANS, anthocyanidin synthase; LAR, leucoanthocyanidin reductase

A schematic flow chat of the phenylpropanoid/flavonoid pathway affected by the chlorosis mutation is shown in Fig. 5. The lower contents were accompanied by the reduced expression levels of most genes (except *CHI* and *F3′H*) involved in the pathway starting from phenylalanine to these metabolites. Furthermore, the expression levels of genes involved in the upstream shikimic acid pathway, which leads to the biosynthesis of phenylalanine, were also strongly down-regulated (Fig. 5). Such findings suggest that flavonoid biosynthesis was strongly inhibited in the chlorotic mutant. One of the explanations for such inhibition lies in the destruction of the chloroplasts, which are the sites of flavonoid biosynthesis [32]. The present work showed that the location of flavonoids in the tissue changed as the leaves became chlorotic, as the anabolic metabolism of flavonoids is coupled with the chloroplast (Fig. 4). Furthermore, the reduced accumulation of flavonoids could be attributed to the low glucose content of the chlorotic mutant. This finding corroborates the findings of Yang et al. [39], who showed that the metabolism of shikimic acid, prephenic acid, and phenylpyruvic acid was strongly inhibited in dark-induced chlorotic tea plants. The decrease of sugars also negatively affected the glycosylation of catechins, as well as the synthesis of flavonoid glycosides [4].

The accumulation of small molecules with antioxidative activity plays important roles in mitigating ROS accumulation [40]. These low-molecular-weight antioxidants include sugars, a-tocopherols, glutathione, amino acids (e.g., proline), ascorbic acid, carotenoids and quinic acid derivatives (e.g., chlorogenic acid). In our study, the tea mutant did not develop functional chloroplasts (data not shown) and lacked chlorophyll under light conditions (Table 1), suggesting that the mutant without shading was exposed to light stress. Many phenylpropanoid [27] and anthocyanin [28] compounds are effective sunlight attenuators and protect photosynthetic organs (chloroplasts) faced with a superabundance of radiant energy. Flavonoids play a vital role in protecting plants to excess UV-B or sunlight irradiance [29–31]. Flavonoids such as quercetin and dihydroxy catechins also serve multiple functions in higher plants under distinct environmental conditions [26]. Flavonoids with a dihydroxy structure in the B-ring accumulate preferentially in response to high doses of ultraviolet radiation (UV) or sunlight radiation [10, 32, 33]. Therefore, the physiological mechanisms in leaves enduring light stress may be associated with dihydroxy flavonoids. In our study, chlorotic leaves contained higher levels of dihydroxyflavonoids, mainly as catechins (catechin gallate, epicatechin) and quercetin and its glycosides, whereas the levels of myricetrin and kaempferol glycoside were lower than in green leaves (Fig. 5, Tables 1 and 2). The similar results have been observed under sun light and partially shading treatments for normal tea plants [5], which suggest that the functions of flavonoids to ROS are not only available in chlorotic tea leaves, but also in all normal tea species. We also found that the dihydroxy flavonoids were mainly distributed intracellularly in the leaf epidermal cells and in the light-receiving area, whereas chlorophyll and catechin were distributed throughout the cells of the chlorotic tea mutant.

Generally, plants possess diverse photoprotection mechanisms, including the dissipation of absorbed light energy as thermal energy by non-photochemical quenching (NPQ). Photoinhibitory quenching (qI), quenching due to state transitions (qT), and high-energy-state-quenching (qE) are the three main components of NPQ, among which qI is related to the slow conversion of the xanthophyll cycle pigment zeaxanthin to violaxanthin, while qE depends on the existence of special xanthophyll molecules (i.e., lutein and zeaxanthin) bound to the PSII antenna proteins [41, 42]. We observed a significant increase in the zeaxanthin and carotene contents in the chlorotic leaves, although the total content of carotenoids decreased by 54% (Table 1). The xanthophyll cycle protects plants from high light intensity by converting violaxanthin into zeaxanthin, which participates in the thermal dissipation of excess absorbed light energy [34]. We also noted higher transcript levels of violaxanthin de-epoxidase and zeaxanthin epoxidase (EC: 1.10.99.3, 1.14.13.90, a key gene in xanthophyll cycle that protects plants from high-intensity light) in chlorotic leaves compared with green (shaded) leaves with transcriptomic analysis (data not shown here). Suggesting that the xanthophyll cycle plays an important role in protecting ‘*Huangjinya*’ from high light intensity. This result is highly consistent with the findings of Li et al. [20]. The tea mutants ‘*White leaf No.1’* and ‘*Huangjinya*’ both showed reduced carotenoid and zeaxanthin content in chlorotic leaves compared with ‘*Fuding dabaicha*’ [17, 43]. Thus, ‘*Huangjinya*’ shows a significant difference in carotenoid levels compared with other tea mutants (mainly the albino mutants). We hypothesized that the variation in carotenoid composition and biosynthesis is a specific light protective mechanism in light-sensitive tea mutants. However, serious damage to cell membrane structures in chlorotic leaves suggests that antioxidants and the xanthophyll cycle were insufficient to protect tea plants from photodamage.

**Fig. 6:**
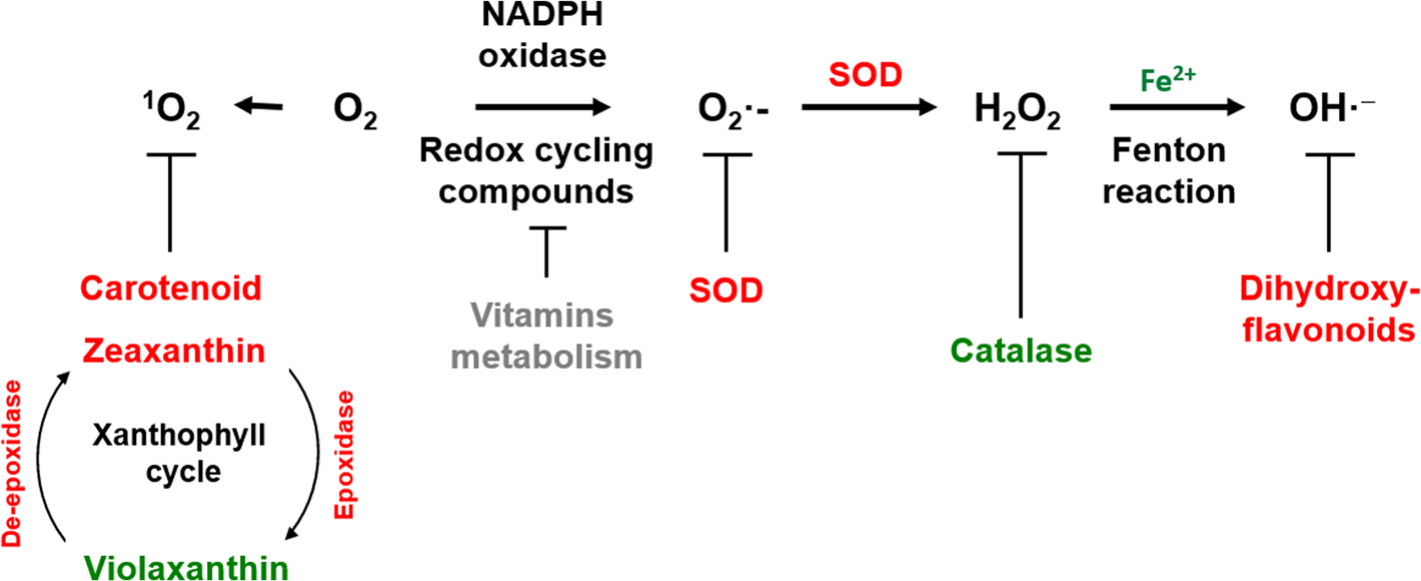
Production and scavenging of free radicals. *Red* and *green* font indicate up- and down-regulated genes / metabolites in the chlorotic leaves compared to shaded plants

Under abiotic stresses, including light stress, highly reactive and toxic ROS such as superoxide anion radicals, singlet oxygen, hydrogen peroxide (H_2_O_2_), and hydroxyl radicals (^•^OH) are induced (Fig. 6), and they are mitigated by ROS scavenging systems [24]. Chloroplasts respond to ROS via a significant change in composition, resulting in rapid morphological and functional modifications [44]. The inactivation of the ROS scavenging system and the biofilm protection function in the chloroplast may cause damage to the membrane structure and the disintegration of chloroplasts. Chloroplasts react violently to ROS generated by high-intensity light [23]. CAT is an iron porphyrin prosthetic group-conjugated enzyme that scavenges ROS [45]. In our study, gene encoding CAT activity down-regulated and iron content decreased (1.6 mg/kg in chlorotic and 1.1 mg/kg in green leaves) in chlorotic leaves. These results suggest that CAT synthesis might be restricted by iron utilization and chelation.

## Conclusions

In this study, the variation in gene expression patterns and metabolites between chlorotic leaves and normal green leaves (leaves under shading treatment) in the ‘*Huangjinya*’ tea mutant was uncovered using metabolomics analysis. Our results suggest that high-intensity light stress caused photooxidation in chlorotic leaves and induced multi-operational photoprotection mechanisms for scavenging ROS, including the activation of dihydroxy flavonoids and xanthophyll cycle pathways, which also reversed the photodamage in mutant leaves, helping them endure light stress. Moreover, the differential accumulation of metabolites and differential gene expression suggested that dihydroxy flavonoids have great differences in terms of their functional roles in tea leaves. Especially, the accumulation and location of quercetin and its glycosides (compare with myricetrin and kaempferol glycoside) in chlorotic leaves suggest their great contribution to photo-protection and their unique functional roles in ROS scavenging in tea leaves.

## Additional file

Additional file 1: Table S1. Primer sequences for quantitative RT-PCR of genes related to the antioxidant system in chlorotic tea leaves. (DOCX 16 kb)

## Abbreviations

^•^OH: hydroxyl radicals
4Cl: 4-coumarate--CoA ligase
ANR: anthocyanidin reductase
ANS: anthocyanidin synthase
C4H: cinnamate 4-hydroxylase
CAT: Catalase
CHI: Chalcone isomerase
CHS: Chalcone synthase
DFR: Dihydroflavonol-4-reductase
F3′5′H: flavonoid 3′, 5′-hydroxylase
F3H: Flavanone 3-hydroxylase
F3′H: Flavonoid 3′-monooxygenase
FLS: Flavonol synthase
H_2_O_2_: Hydrogen peroxide
HPLC: High-performance liquid chromatography
LAR: Leucoanthocyanidin reductase
NPQ: Non-photochemical quenching
PAL: Phenylalanine ammonia-lyase
PLS-DA: Projection to latent structure discriminant analysis
PSII: *Photosystem II*
qE: High-energy-state-quenching
qI: Photoinhibitory quenching
qRT-PCR: Quantitative real-time PCR
qT: Quenching due to state transitions
ROS: Reactive oxygen species
SOD: Superoxide dismutase
UPLC-Q-TOF/MS: Ultra-performance liquid chromatography quadrupole time-of-flight mass spectrometry
UV: Ultraviolet radiation
VIP: Variable importance in the projection
