# Supplementary material for: Metabolomics analysis reveals the metabolic and functional roles of flavonoids in light-sensitive tea leaves
Source: BMC Plant Biol. 2017 Mar 20;17:64. doi: 10.1186/s12870-017-1012-8 (PMC5359985; doi:10.1186/s12870-017-1012-8)
Supplement: Additional file 1: Table S1. — Primer sequences for quantitative RT-PCR of genes related to the antioxidant system in chlorotic tea leaves. (DOCX 16 kb) [file 12870_2017_1012_MOESM1_ESM.docx]

Additional file 1: Table S1: Primer sequences for quantitative RT-PCR of genes related to the antioxidant system in chlorotic tea leaves.

| **Genes** | **Forward Primer (5’ - 3’)** | **Reverse Primer (5’- 3’)** |
| --- | --- | --- |
| 4CL | CGTGGTCCTCAAATTATG | CATCATCGTCGTCTACAT |
| ANR | GCGAAGTTGATCCTCTCGTC | AACCACATCGTCAAGTGAACA |
| ANS | TAATGGCAAGTACAAGAG | CAATGGCTTCAAGATAATC |
| C4H | CAATGGCAATGACTTTAG | CTCAGCAGTATCAATCTT |
| CHI | GTGATGGATGAAGTTGTG | AAGAGAGAAAGCAGAGTC |
| CHS | TTACTAATAGCGAGCATAAGGT | CTAGCATCAAGCGAAGGT |
| DFR | AGTTGTGTCGTTCTCATC | GTATCAATGGCTCCTCTG |
| F3’5’H | AATCCTGGTGAAGAGAAG | TCTATTATGCTTGATGATGTG |
| F3’H | ACCTTTCGACTTCACCCATCAAC | TAACTGGACCATACGCAACCCTA |
| F3H | GCGACAGTATACCCCCTGAA | AGTATGGCAAAGGCACATCC |
| FLS | GGAGAACAGCAAGGATATCG | TCTCCTCCTGTGGGAGCTTA |
| LAR | GGGGCATCCTGTATCAAAGA | CCGCATACCTTTCAGTCCAT |
| PAL | ATGACTTCTACAACAATGG | GGAGTTCTGAGCAATAAG |
| SOD | CATTTCAATCCTGCTGGCAAAGA | GCATGGACAACAACGGCCCTACC |
| CAT | TGCAGAGAATGAGCAGCTTG | GTGCCTCTGGGTATCAGCGTAG |
| GAPDH | TTGGCATCGTTGAGGGTCT | CAGTGGGAACACGGAAAGC |
